# Care for Social Isolation and Loneliness in a Case With Late-Onset Delusional Disorder

**DOI:** 10.7759/cureus.56697

**Published:** 2024-03-22

**Authors:** Hideki Kanemoto, Tatsuhito Kawasaki

**Affiliations:** 1 Department of Psychiatry, Osaka University Graduate School of Medicine, Osaka, JPN; 2 Department of Psychiatry, Mizuma Hospital, Osaka, JPN

**Keywords:** chronic pain, daycare service, non-pharmacological treatment, group occupational therapy, cognitive therapy, supportive psychotherapy, loneliness, social isolation, late-onset delusional disorder

## Abstract

Late-onset psychosis refers to the development of psychotic symptoms after the age of 40 and can encompass various conditions like schizophrenia, mood disorders with psychotic features, and delusional disorder. Non-pharmacological interventions are critically important in older adults with psychosis, especially considering the lack of evidence for the efficacy of antipsychotics and the high risk of side effects. Social isolation is recognized as one of the risks of late-onset psychosis, and interventions to eliminate this risk are becoming increasingly important in Japan, where social isolation among older people is widespread as society ages. We present a case of late-onset delusional disorder in which multidimensional interventions for social isolation and loneliness, which have significant impacts on symptoms, were effective in achieving persistent remission. The case involved a woman in her mid-70s who began to complain of paranoia that taking a painkiller recommended by her husband caused persistent pain. Her husband’s hospitalization and the deterioration of her relationship with her children resulted in her becoming socially isolated, which intensified her delusions and led to aggressive behavior and depression. Although antipsychotic medications had a limited effect, remission was achieved through supportive psychotherapy, cognitive therapy, family education, participation in group occupational therapy, and the introduction of daycare services, which were implemented to reduce loneliness and social isolation. Specifically, supportive psychotherapy and family education for her loneliness, awareness of another possible cause of pain that she developed through Socratic questioning, and environmental adjustments played important roles in preparing her to accept cognitive therapy, achieve delusional remission, and maintain her state of remission, respectively. The interventions implemented in this case provide insights for addressing social isolation and loneliness in late-onset psychosis.

## Introduction

Late-onset psychosis refers to the development of psychotic symptoms after the age of 40 and can encompass various conditions like schizophrenia, mood disorders with psychotic features, and delusional disorder. Late-onset psychosis, similar to younger-onset psychosis, is characterized by strong delusions and hallucinations, mainly persecutive paranoia. However, late-onset psychosis differs from young-onset psychosis in that negative symptoms and thinking disturbances are rarely prominent. In 2000, the international late-onset schizophrenia group defined late-onset schizophrenia, which develops after the age of 40 years, and very-late-onset schizophrenia-like psychosis (VLOSLP), which develops after the age of 60 years, separately from typical-onset schizophrenia spectrum disorders [1].

Similar to younger-onset psychosis, late-onset psychosis is generally treated with antipsychotics. However, the high risk of adverse effects of antipsychotics in older adults and the lack of evidence for the efficacy of antipsychotics in older-onset cases often makes pharmacological treatment of late-onset cases difficult [2]. Therefore, non-pharmacological treatment is important for late-onset psychosis.

Social isolation has been reported as a pathogenic factor in late-onset psychosis [3], and non-pharmacological interventions targeting social isolation may be effective in the treatment and relapse prevention of late-onset psychosis. As society ages and the number of older adults living alone increases, there is an increasing need for such interventions. However, there is no clear evidence for such non-pharmacological interventions. In this case report, we describe a patient with late-onset psychosis who had been socially isolated and went into persistent remission even after discontinuation of antipsychotics because of multidimensional interventions to reduce her loneliness and social isolation. The patient provided her written informed consent to submit this case report. This case report was prepared in compliance with the CARE guidelines.

## Case presentation

The patient was a woman in her mid-70s. She had no birth or developmental abnormalities, had worked for several years after graduating from junior high school, married in her 20s, and became a housewife. She had two children and lived with her husband for many years. Their children lived nearby and visited them several times weekly. She was always sociable and often went out with friends. However, as she aged, her social interactions with her friends decreased. The patient had no history of psychiatric visits.

She complained of headaches seven months prior, and her husband recommended that she take acetaminophen. From approximately the same time, severe pain localized in the right oral cavity persisted. She thought the pain was caused by the acetaminophen she took and began to complain to her husband, saying, “The pain happened because you made me take the drug,” in a paranoid manner. She was examined at several hospitals for pain; however, no apparent organ abnormalities were observed. She continued to complain of pain, but her family gradually discontinued acceptance of her complaints.

Her husband was hospitalized for heart disease four months earlier; hence, she was left alone. Her children became busy visiting her husband and had fewer opportunities to see her. Since then, her paranoid complaints targeting her husband intensified, and she began exhibiting behavioral problems, including calling her children day and night, complaining persistently of oral pain, resentment towards her husband, and the unusual experience of “worms coming out of my mouth,” calling her children in the middle of the night to tell them that she was going to die, and locking herself in her house, prompting police intervention.

After her husband was discharged from the hospital one month prior, she continued to complain of pain and paranoia and became verbally abusive and violent towards him. Her family brought her to our hospital, seeking treatment for delusions and behavioral abnormalities. At the beginning of the examination, she responded to the doctor politely and calmly, saying that she had no problems except for the pain in her mouth. However, when asked for details about her pain, she replied, “It has been hurting ever since I took the medicine that my husband told me to take. My husband made me take it to make me suffer” and showed hostility towards her husband. Paranoia and anger were evident, and she was admitted to our hospital for further examination and treatment.

No abnormal neurological findings were observed, including Parkinsonism. A general physical examination revealed no abnormalities. Electroencephalography, electrocardiography, chest radiography, and routine laboratory tests revealed no abnormalities. Cranial computed tomography revealed a slight global cerebral atrophy, that was considered age appropriate. The Mini-Mental State Examination (MMSE) score was 24 out of 30, with one point deducted each for time and place orientation and four points for serial sevens. These results show that, in terms of cognitive function, the patient had attention impairment, but other cognitive domains, including memory, were preserved. The patient was independent in activities of daily living. The persistent pain in the right corner of her mouth and delusions regarding her husband persisted even after admission. She was diagnosed with a delusional disorder and chronic pain caused by an organic cause.

Following admission, she exhibited agitation, aggression, and a mild depressive mood, in addition to delusions. She expressed suicidal ideation and was seen strongly appealing to the doctor from the top of the stairs, saying, “I am going to jump down from here.” To manage the behaviors associated with her agitation and depressive mood, she was isolated in a protective room and prescribed 1 mg/day brexpiprazole for antipsychotic and sedative effects. Her symptoms gradually improved, and she was able to spend time in the ward with other patients within a few days. Although agitation, aggression, and suicidal ideation disappeared, delusions regarding her husband, depressive mood, and chronic pain persisted.

The events associated with her symptoms and her husband’s hospitalization made her feel alienated from her family and increased her loneliness, which was thought to be one of the factors that strengthened her delusions. Therefore, from the earliest possible stage, we encouraged her children to visit her actively. Additionally, she was encouraged to participate in group occupational therapy in the ward.

To identify the causes of pain and resolve her delusional interpretations of pain, her attending psychiatrist initiated supportive psychotherapy for 30 min to 1 h per day. When interviewed in detail regarding the nature of the pain, she spontaneously stated that the pain, that was localized only from the right corner of the mouth to the right lower jaw, started after taking acetaminophen seven months ago and that she had not taken acetaminophen again since then. Her attending psychiatrist supported her in acknowledging that although the physical examination did not identify any abnormalities that could be the cause of the pain, he believed that she felt pain and wanted to identify the cause of the pain. When asked why she took acetaminophen, a painkiller, she admitted that she had experienced pain before taking it. The psychiatrist then told her that it was unlikely that the side effects would persist even though the medication had been discontinued for seven months and that the side effects of oral medication could be systemic rather than localized symptoms. Over the course of these sessions, she gradually began to doubt that the medication caused the pain. After one week, when asked about potential factors related to the pain in the right corner of her mouth, she spontaneously mentioned recurrent herpes blisters that coincided with the painful areas, which was the dominant region of the third branch of the trigeminal nerve. We confirmed that the patient tested positive for herpes simplex antibodies and reported this finding to her. Subsequently, she came to interpret the sore mouth as being caused by herpes rather than the medication recommended by her husband. Thus, her paranoia targeting her husband and her depressive mood disappeared gradually. It remained uncertain whether the pain was due to herpes; however, considering the possibility of neuropathic pain, pregabalin (150 mg/day) was prescribed. Subsequently, the pain fluctuated but gradually decreased.

**Figure 1:**
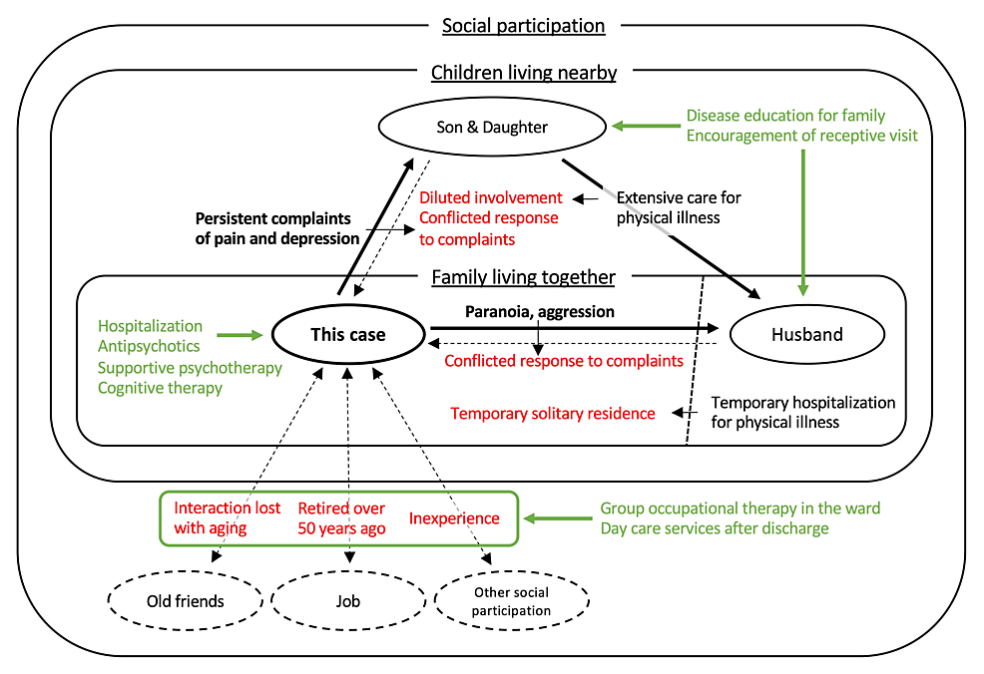
Multidimensional social isolation and loneliness in this case. Bold, symptoms of this case. Red, factors of her social isolation/loneliness. Green, interventions for her symptoms and social isolation/loneliness.

We determined that psychosocial factors such as social isolation and loneliness were also involved in her delusions (Figure 1). Active participation in group occupational therapy in the ward was encouraged, and the patient appeared to enjoy the program while interacting with other patients. Disease education was provided to her family members, encouraging them to meet and be receptive to her. After confirming that her dissatisfaction with her husband had been resolved, she met her husband and was able to spend time with him in a cheerful manner. Even during a temporary stay at her home, she had a peaceful time with her husband. She was initially reluctant when told that regular daycare services were desirable upon discharge. However, she agreed after learning that the daycare program was similar to the group occupational therapy program she had participated in in the ward. After arranging daycare services, she was discharged after approximately 40 days.

In the outpatient clinic after discharge, although she occasionally complained of pain, she did not experience any relapse of delusions and reported to her psychiatrist that she was enjoying daycare services with her new friends. Approximately seven months after starting oral administration of brexpiprazole, she began to exhibit small steps, magnetic gait, and instability during walking. Her overall movements also slowed, and she reported falling at home. No abnormalities in the muscle tone or involuntary movements, including tremors, were observed. However, it was judged to be drug-induced Parkinsonism, and the prescription of brexpiprazole was discontinued. Since then, her walking ability has improved, delusions have not recurred, and she has been able to maintain good relationships with her family members and friends. Approximately 8 months after discharge, her mental state remained stable, and her MMSE score was 25 points, with no change in cognition compared to that at the initial visit.

## Discussion

This patient, in her mid-70s, had persistent delusions for more than six months and began to experience oral pain under the influence of oral medications recommended by her husband, with no evidence of coherent speech, catatonic, or negative symptoms. If oral pain was a hallucination, it was strongly related to the delusion of the patient and did not present with any other hallucinations. Although the patient was depressed and initially had suicidal ideation, she did not meet the criteria for a major depressive episode and had no history of manic or depressive episodes. MMSE and her life on the ward during hospitalization revealed that she had mild attention impairment but generally maintained cognitive function, was independent in basic activities of daily living, and did not present with dementia. No physical illness or substance use was identified as the cause of psychosis. Based on the above, she met the diagnostic criteria for delusional disorder in the DSM-5 and was classified as a persecutory type or perhaps a mixed type with persecutory and somatic delusional themes. The following factors contributed to remission in this case: (1) pharmacotherapy with antipsychotic drugs to improve her behavior related to delusions, agitation, and depressive mood in the acute phase; (2) modifying delusional interpretations of pain through supportive and cognitive psychotherapy; (3) elimination of loneliness by promoting family acceptance through disease education; and (4) group occupational therapy in the ward and introduction of daycare services after discharge to eliminate her social isolation.

Upon admission to the hospital, she experienced severe paranoia and agitation, accompanied by a depressive mood with suicidal ideation, making psychotherapy and occupational therapy difficult. Therefore, pharmacological treatment with an antipsychotic was prioritized. There is limited high-quality evidence on the use of antipsychotics for late-onset psychosis. A randomized controlled trial of VLOSLP, including late-onset delusional disorder, demonstrated the effectiveness of low-dose amisulpride [4]. Brexpiprazole, which is effective in schizophrenia [5], is also expected to influence emotional stability. Therefore, brexpiprazole was selected for its effects on depression, agitation, and delusions in the present case. Brexpiprazole was effective against depression and agitation but not against delusions in the acute phase.

However, the long-term use of antipsychotics in older patients is associated with a high risk of side effects, including extrapyramidal symptoms [2], as observed in this case. This is due to increased sensitivity resulting from age-related pharmacokinetic and pharmacodynamic changes [6]. In addition, it has been suggested that a certain percentage of patients with late-onset psychosis have Lewy body disease neuropathology [7]. Since Lewy body disease is characterized by hypersensitivity to antipsychotic medications [8], it may contribute to the high risk of antipsychotic adverse effects in patients with late-onset psychosis. In cases such as the present one, careful follow-up is crucial to monitor for potential conversion to DLB in the future. Specifically, the patient temporarily made statements suspicious of a delusional infestation. Moreover, given that there have been cases of DLB with delusional infestation as an early symptom [9], it encourages careful follow-up.

In this case, the patient remained in remission after discontinuation of brexpiprazole owing to side effects. This may be attributed to the non-pharmacological interventions for social isolation and loneliness used to treat psychosis and depressive mood that remained after brexpiprazole treatment and to prevent psychotic relapse. This suggests that environmental factors, such as social isolation and loneliness, play a crucial role in the development of late-onset psychosis, similar to late paraphrenia and Janzarik’s kontaktmangelparanoid [3]. These factors are still considered risk factors for the development of VLOSLP [1]. In the present case, various levels of social isolation may have influenced the onset of delusions at an older age. While the patient repeatedly complained of pain, the examination conducted did not reveal any organic abnormalities as the cause. Gradually, her family experienced difficulties in dealing with her complaints. Families of patients with chronic pain often report that they feel that patients appear to complain more strongly about their pain than they are aware of [10]. Owing to these conflicts with family members, patients with chronic pain tend to have a low sense of family bonding and solidarity, which is reportedly associated with psychological distress [11]. Similarly, in the present case, the experience of chronic pain led to feelings of loneliness due to a conflict with her family.

The patient had always been sociable, but as she aged, her friendships decreased. Additionally, just before her delusions became severe, her children’s attention was no longer focused on her because of her husband’s physical illness, which deepened her isolation from the family. Her social isolation was further reinforced, and she felt lonely. The initial non-pharmacological therapy aimed at relieving her loneliness consisted of supportive psychotherapy for pain by her attending psychiatrist, education about her illness, advice to her family to behave acceptably towards her complaints of pain, and repeated receptive visits by her children. The decisive event that improved her paranoia was that she came to consider another possible cause of the pain through Socratic questioning during psychotherapy. Some previous reports have suggested that supportive psychotherapy is helpful for most patients and that cognitive therapy, which primarily involves the replacement of maladaptive thoughts with more adaptive beliefs using Socratic questioning, has been effective in the treatment of delusional disorders [12] while being careful to avoid hasty coping in the acute phase. Thus, psychotherapy played a major role in the remission of psychosis in this case.

Some previous studies have employed a five-component scale (partner, children, relatives, friends, and social organization) to assess social isolation among older adults. One of these studies has shown that mental health issues, including depression, were related to social isolation, especially poor interaction with children and friends, and a lack of social participation in Japan [13]. In the present case, the concentration of her children’s attention on her husband and the dilution of her past friendships would have had a significant impact on her mental state. The number of older people living alone is increasing along with the aging of society in Japan, and social isolation among older people has reportedly increased in 2016 compared with 2010 in Japan; the social isolation score increased by 7.3% for men aged 65 to 74, 6.3% for women aged 65 to 74, and 3.2% for women aged 75 and older [14]. It is anticipated that mental health issues, such as those in this case, will increase in Japan and around the world.

Given these considerations, we believe that the introduction of daycare services and the family therapy described above for her social isolation played an important role in maintaining remission after discharge from the hospital, as suggested in a previous report [3]. Such outpatient services are expected to be effective for delusions in patients with dementia [15]. However, she was initially reluctant to use daycare services, saying, “I do not know what kind of things I will be doing there.” However, during her hospitalization, she showed smooth and frequent interactions with other patients, frequently participated in daily group occupational therapy, and was satisfied with the program. After informing her that daycare services offer activities similar to those in her current ward life, requesting her care manager select services that incorporate more group therapy, including recreation, and sharing information with her regarding potential daycare services, she gradually became more open to introducing these services. In Japan, daycare services have been offering various activities to improve the quality of life of older adults, support their independence, and reduce the burden on family caregivers since the introduction of the long-term care insurance system in 2000. However, many older adults refuse to start using daycare services. To facilitate the acceptance of daycare services by individuals who are reluctant, with minimum resistance, the following factors are required: (1) respecting their thoughts and feelings; (2) providing opportunities for them to learn about the services; (3) considering and implementing service programs that meet their needs; (4) establishing and maintaining the human and physical environment for services; and (5) cooperating and working with family and other related persons [16]. The process of acceptance of daycare services observed in this case was based on the establishment of these factors. Participation in group occupational therapy was particularly helpful in facilitating the patient’s understanding and acceptance of the services in a tangible manner.

She visited our hospital relatively smoothly because she lived with her husband, her children lived in the neighborhood, and because the pain was severe. However, patients with late-onset psychosis, which tends to occur in social isolation, rarely voluntarily visit medical institutions because they have little awareness of their illness and can maintain their ability to live because of a lack of negative symptoms or thought disorders despite strong delusions. In Japan, the Initial-Phase Intensive Support Team for Dementia, a multidisciplinary outreach team that provides initial assessment and support for dementia, often encounters complex cases due to behavioral and psychological symptoms, including psychosis [17]. In such cases, the teams sometimes encounter cases with late-onset psychosis, which presents challenges for the community. Efforts to encourage earlier consultation among patients with late-onset psychosis or prevent the onset of late-onset psychosis are expected to expand. Regarding initiatives to prevent social isolation among older adults, information and communication technologies are being used to reduce loneliness among older adults living alone [18,19]. Telecommunications technologies, prevalent during the COVID-19 pandemic, may also have a positive impact on the social functioning of older adults [20]. These technologies are expected to expand efforts aimed at the prevention and early detection of late-onset psychosis.

## Conclusions

Rapid changes in the social structure in Japan may increase the risk of developing late-onset psychosis, which may be influenced by social isolation and loneliness, as observed in this case. In addition to pharmacological therapy, appropriate psychosocial approaches for loneliness and social isolation are beneficial for the treatment and prevention of late-onset psychosis. In the present case, the supportive psychotherapy and family education for her loneliness, the awareness of another possible cause of pain that she came to through Socratic questioning, and the environmental adjustments played important roles in putting her in a state to accept cognitive therapy, her delusional remission, and maintaining her state of remission, respectively. However, these initiatives require significant resources. Thus, implementing these initiatives on a wide scale remains a challenge for the future.
